# Characterization of cold sensitivity and thermal preference using an operant orofacial assay

**DOI:** 10.1186/1744-8069-2-37

**Published:** 2006-12-13

**Authors:** Heather L Rossi, Charles J Vierck, Robert M Caudle, John K Neubert

**Affiliations:** 1 College of Dentistry Department of Orthodontics, University of Florida, 1600 S.W. Archer Road, P.O. Box 100444, Gainesville, FL 32610-0444, USA; 2 College of Dentistry Department of Oral Surgery, University of Florida, 1600 S.W. Archer Road, P.O. Box 100416, Gainesville, FL 32610-0444, USA; 3 College of Medicine Department of Neuroscience, University of Florida, 100 Newell DR., P.O. Box 100015, Gainesville, FL 32610-0444, USA; 4 Evelyn F. and William L. McKnight Brain Institute, University of Florida, 100 Newell DR., P.O. Box 100015, Gainesville, FL 32610-0444, USA

## Abstract

**Background:**

A hallmark of many orofacial pain disorders is cold sensitivity, but relative to heat-related pain, mechanisms of cold perception and the development of cold allodynia are not clearly understood. Molecular mediators of cold sensation such as TRPM8 have been recently identified and characterized using in vitro studies. In this study we characterized operant behavior with respect to individually presented cold stimuli (24, 10, 2, and -4°C) and in a thermal preference task where rats chose between -4 and 48°C stimulation. We also evaluated the effects of menthol, a TRPM8 agonist, on operant responses to cold stimulation (24, 10, and -4°C). Male and female rats were trained to drink sweetened milk while pressing their shaved faces against a thermode. This presents a conflict paradigm between milk reward and thermal stimulation.

**Results:**

We demonstrated that the cold stimulus response function was modest compared to heat. There was a significant effect of temperature on facial (stimulus) contacts, the ratio of licking contacts to stimulus contacts, and the stimulus duration/contact ratio. Males and females differed only in their facial contacts at 10°C. In the preference task, males preferred 48°C to -4°C, despite the fact that 48°C and -4°C were equally painful as based on their reward/stimulus and duration/contact ratios. We were able to induce hypersensitivity to cold using menthol at 10°C, but not at 24 or -4°C.

**Conclusion:**

Our results indicate a strong role for an affective component in processing of cold stimuli, more so than for heat, which is in concordance with human psychophysical findings. The induction of allodynia with menthol provides a model for cold allodynia. This study provides the basis for future studies involving orofacial pain and analgesics, and is translatable to the human experience.

## Background

A hallmark of many orofacial pain disorders is cold sensitivity, but unlike heat-related pain, the mechanisms underlying cold perception and the development of cold hypersensitivity are less clearly understood. Most cold research has focused primarily on *in vitro *or immunohistochemical studies; in contrast to heat pain, few behavioral assays relating to cold are available. The hindpaw acetone cooling assay [1,2] has been used to evaluate cold allodynia, but this test is difficult to quantify in terms of the temperature delivered, and it is difficult to separate the mechanical sensation of wetness from the onset of skin cooling. Recently, Allchorne *et al*. demonstrated a stimulus-response function to cold stimulation using a peltier device and recorded hindpaw withdrawal latency as the outcome measure in freely moving animals [3]. This method is more quantifiable than the acetone assay and does not involve restraint-related stress; however, this reflex-based assay provides limited information regarding evaluation of the central processing of pain. In contrast, use of operant assays allows for a more complete assessment of these aspects of pain processing. Mauderli *et al*. [4] and Vierck *et al*. [5] assessed escape latency and duration in an operant assay and found that escape duration revealed stimulus-response relationships better than latency, and the animals were more sensitive to increasing heat than to increasing cold for stimulation of the plantar surfaces of the paws.

We previously developed an operant behavior system to evaluate thermal sensitivity in the face [6]. This assay provides a useful means of evaluating animal models of trigeminal pain in a manner that is relevant and translatable to humans. The objectives of this study were to characterize operant behavior with respect to cold stimuli applied to the face and to evaluate the effects of menthol on that behavior. Menthol is a modulator of cold sensation [7-9], and it stimulates the TRPM8 receptor [10,11]. TRPM8, a member of the transient receptor potential (TRP) receptor family, is a molecular mediator involved in cold sensation and is activated by temperatures below 25°C [10,11]. We hypothesized that increasingly cold stimuli would affect behavioral outcome measures in a manner similar to stimulation of the paws and that application of menthol would enhance cold-induced pain.

Here we substantiated the stimulus response curve for cold and found that it was modest relative to heat on the face. However, the thermal preference paradigm indicated that cold is especially aversive, compared to a level of heat stimulation that produced comparable avoidance behavior. The importance of describing a stimulus-response function was revealed by tests following application of menthol, which enhanced avoidance only for 10°C stimulation. Using menthol in the presence of a cool stimulus appears to provide a model of thermal allodynia analogous to capsaicin-induced hypersensitivity to heat, and taken together with the -4°C stimulus, allows for further translatable studies regarding cold pain.

## Results

### Behavioral response to cold and the effects of sex

In order to characterize normal behavioral responses of male (n = 6) and female (n = 6) rats, we tested them in the cold to neutral range (-4 to 37°C). During training and in recorded sessions at 37°C, there was no trend observed for either sex to consume more milk reward or to satiate at different rates. Table 1 shows the differences (male – female) in raw outcome measures at each temperature tested. For cold temperatures above freezing (24, 10 and 2°C), the males licked less often and consumed less milk reward than females, although these differences were not significant (Table 1). The male-female differences reached statistical significance only for 10°C stimulation, which induced significantly more thermode contacts by males. Also, the ratio of licks to thermode contacts was significantly reduced for 10°C stimulation of males. Thus, particularly for mildly aversive (10°C) cold stimulation, males were hindered from completing the task, as revealed by less licking and more stimulus contacts that did not progress to licking.

**Table 1:** Comparison of behavioral outcomes for males and females.

Outcome Measure	Intake (grams)		Licking Events (count)		Facial contact Events (count)		Duration (seconds)		Licks/Facial contacts ratio		Duration (s)/facial contacts ratio	
Temp.	♂-♀	t	♂-♀	t	♂-♀	t	♂-♀	t	♂-♀	t	♂-♀	t

37°C	1.4	0.63	-110	0.27	11	0.28	162	2.11	7.68	0.63	2.23	1.16
24°C	-1.2	-0.75	-477	-1.33	41	0.95	-23	-0.45	-4.42	-1.16	-0.48	-0.75
10°C	-2.4	-2.05	-341	-1.01	89	2.53*	21	0.45	-6.99	-2.22*	-0.82	-1.74
2°C	-2.2	-0.95	-662	-1.19	19	0.36	216	1.44	-5.07	-0.69	0.97	0.75
-4°C	1.3	0.52	111	0.33	15	0.34	44	0.7	-2.96	1.22	-0.47	-0.87

Shown are mean difference between males and females and the corresponding t statistic for the six outcome measures (intake, licking events, stimulus contacts, duration, licks to stimulus contacts, and stimulus contact duration) across a range of temperatures (37, 24, 10, 2, and -4C). * indicates a significant difference between males and females (P < 0.05).

**Figure 1:**
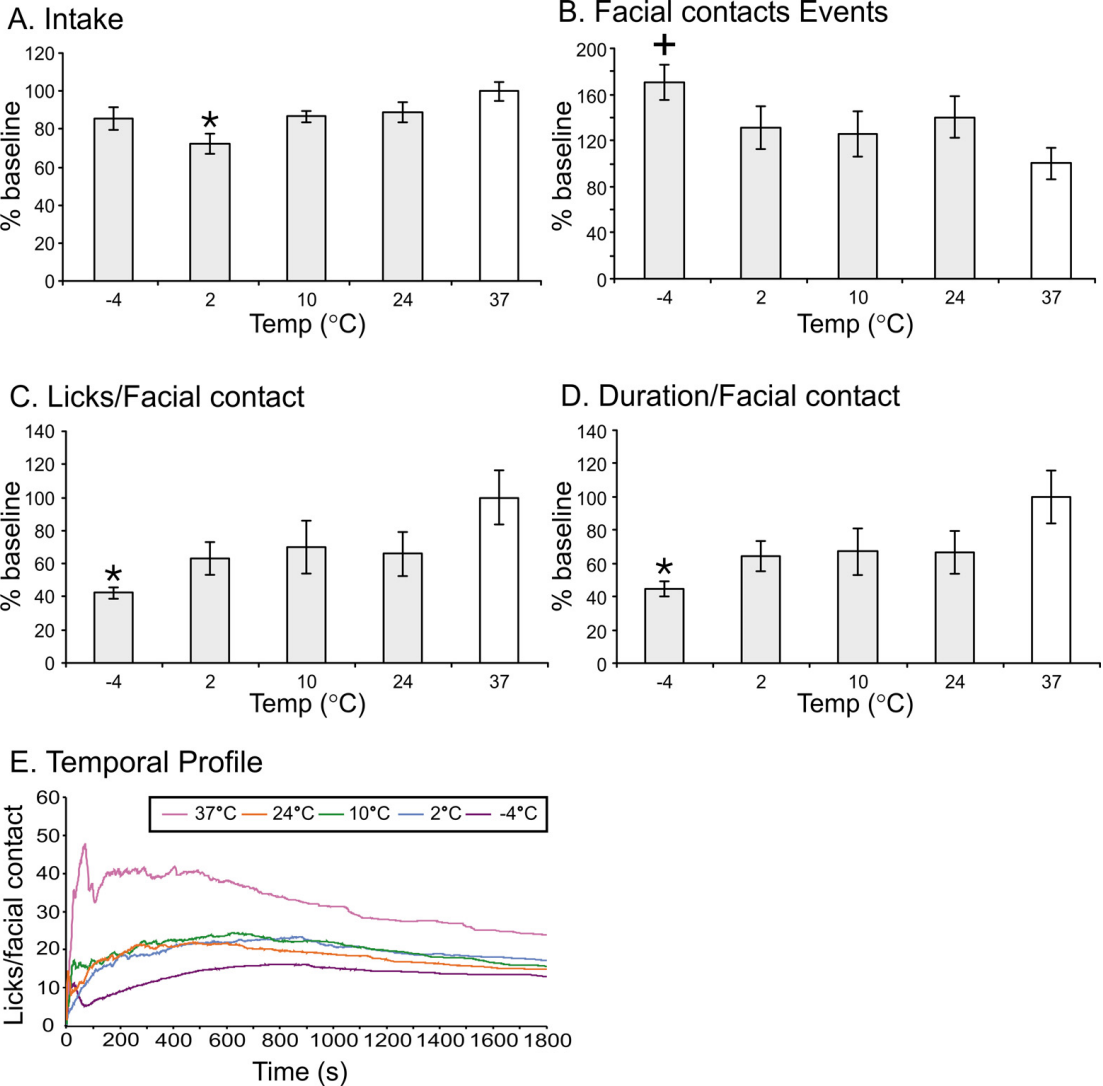
**Behavioral response to cold stimuli**. Four outcome measures are shown: intake (A), facial contact events (B), licks (reward)/facial contact ratio (measures success) (C), and facial duration/contact (D) for cold temperatures (-4, 2, 10, and 24°C), expressed as a percent of baseline (37°C). + indicates significant increase, and * indicates a significant decrease (P < 0.05). (E) represents the change in the licks/facial contact (success ratio) over the 30-minute test period for each of the temperatures tested.

For males and females combined, there was a significant effect of temperature on facial (stimulus) contacts (F_4,71 _= 2.503), the reward/stimulus ratio (F_4,71 _= 4.078), the stimulus duration/contact (F_4,71 _= 4.034), and intake (F_4,71 _= 2.590). Across the range of temperatures presented, increasing cold gradually reduced food intake, the number of licks per contact and the duration of individual contacts (Fig. 1A). Each of these measures depends upon secure contact with the thermode as the animal feeds. In contrast, the number of thermode contacts increased with increasing cold, as the animals more frequently withdrew from the thermode before contacting the feeding tube (Fig. 1B). Post-hoc analyses revealed that the stimulus contacts were significantly increased (Fig. 1B) and both the reward/stimulus ratio and the stimulus duration/contact were significantly lower for -4°C relative to 37°C (Fig. 1C, D). There were no significant differences between -4°C and 2, 10, or 24°C. Additionally, there was no significant effect of temperature on the total number of licking events or total duration of facial contacts, and there was no significant effect due to estrus stage of females (*data not shown*).

Sensitivity to cold can change over time due to factors such as changes in blood flow and receptor adaptation. Therefore we looked at changes in the ratio of licks to facial contacts over the 30-min trial. Fig. 1E shows changes in the licks to facial contact ratio over time for 37, 24, 10, 2, and -4°C. There was a clear separation between 37°C and the cool and cold temperature trials Throughout the 30-minute period, 37°C produced the greatest licks to facial contact ratio over time, while -4°C produced the lowest licks to facial contact ratio over time, and the 2, 10 and 24°C produced intermediate response profiles. Note that the animals attained their maximal rate of licks per contact early in the 37°C stimulation. After a sharp peak of licking probably related to hunger, a high rate of licks per contact was maintained for approximately 8 minutes, followed by a very gradual reduction as satiation progressed without cold stimulation. In contrast, the maximum ratios of licking per contact during cold stimulation were attained in excess of 10 minutes into the trials. This suggests that the rate of licking per contact increased as cold adaptation progressed within trials.

### Behavioral response to thermal preference

**Figure 2:**
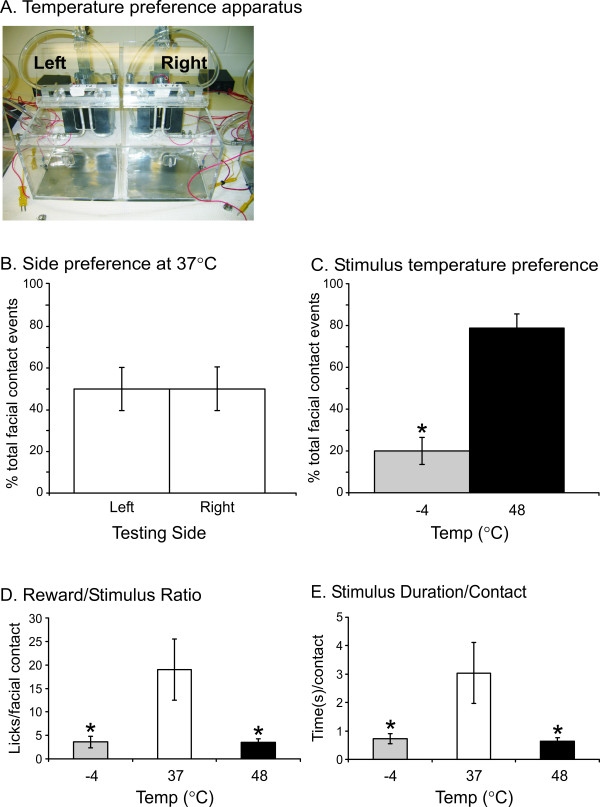
**Behavioral response to thermal preference**. Rats were first placed in the thermal preference testing box (A) with left and right thermodes set at 37°C to ensure that there was not a side preference (B). In two additional testing sessions, rats chose between -4 and 48°C (C) and their reward/stimulus (D) and stimulus duration/contact ratios were found to be no different for -4°C and 48°C, but significantly lower for -4°C and 48°C relative to 37°C (ratios calculated from temperature choice trials). * indicates significant decrease, P < 0.05.

A separate group (n = 7) of male rats was first tested under neutral conditions (37 °C) on both sides of the place preference box (Fig. 2A). There were no significant differences in the percent of total intake, licks, facial contacts, or duration between the left and right sides at 37°C (Fig. 2B – *facial contacts shown*), indicating that the rats did not have a side preference. Rats switched sides an average of seven times. When presented with a choice between -4 and 48°C stimulation, the percentage of licks (t_13 _= -2.98, *P < 0.05), facial contacts (t_13 _= -6.05, P < 0.001), and duration (t_13 _= -3.22, P < 0.05) was greater for the hot thermode than the cold, regardless of the side assignment for each temperature (Fig. 2C – *facial contacts shown*). The rats switched sides an average of six and seven times for each of two trials; thus there wasn't a decrease in exploration that could account for a lack of activity on one side or the other. The licks per thermode contact and duration per thermode contact in the thermal preference tests were also calculated for 37, 48, and -4°C as a comparison of the relative amount of pain at each of these temperatures (Fig. 2D, E). These ratios were calculated using data obtained in the preference condition, not in single-temperature trials. Both of these ratios for 48 and -4°C were significantly different than those for 37°C (licks/facial contact F_2,20 _= 5.40, duration/facial contact F_2,20 _= 4.63, P < 0.05). However, there was no significant difference between 48 and -4°C for either ratio. Thus, the rats expressed a greater aversion for -4°C cold, or conversely, a preference for 48°C heat, even though success performance at the two thermodes was comparable.

### Behavioral response to menthol and vehicle treatment

**Figure 3:**
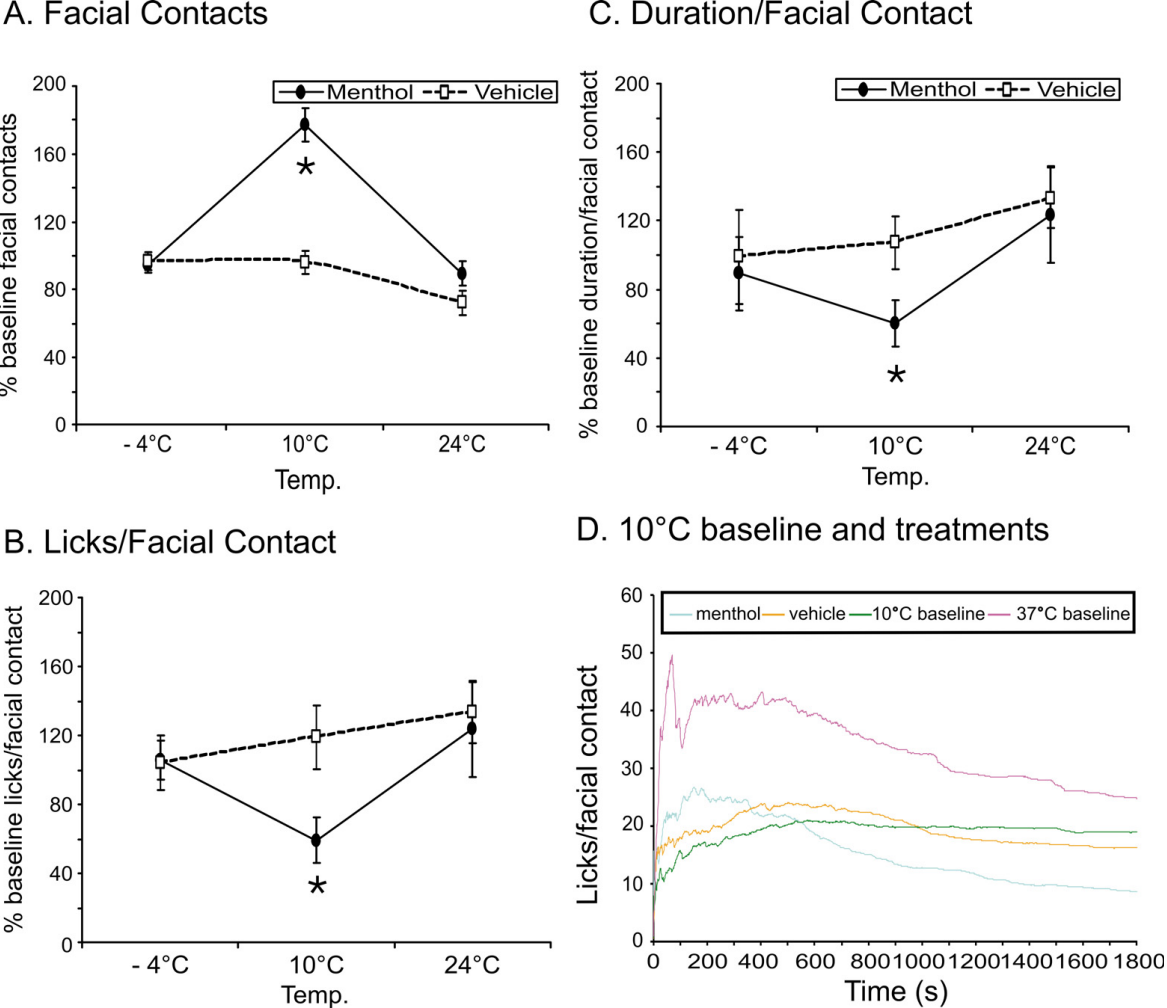
**Behavioral response to menthol and vehicle treatment**. Stimulus contacts (A), reward/stimulus success ratio (B), and stimulus duration/contact (C) at 24, 10, and -4°C in the presence of menthol (solid lines) or vehicle (dotted lines), expressed as a percent change from untreated baseline temperatures. Data points indicate average change from baseline, + indicates significant increase, and * indicates a significant decrease. The change in reward/stimulus success ratio over time (D) is also given for menthol (blue), vehicle (orange), baseline at 10°C (green), and baseline at 37°C (pink).

Rats were tested at 24, 10, and -4°C fifteen minutes following injection of either menthol or vehicle. Each individual rat's outcome measures were compared to the baseline average for its sex to produce a percent increase or decrease from baseline for the menthol and vehicle treatment groups. At 10°C, there was a significant treatment effect on total facial contacts (F_2,35 _= 8.582), the ratio of licks to facial contacts (F_2,35 _= 5.476), and the duration of facial contact (F_2,35 _= 4.640) (Fig. 3A–C), when comparing menthol with vehicle. Following menthol treatment, total facial contacts increased relative to vehicle treatment and baseline at 10°C. The ratio of licks to facial contacts and the duration per facial contact decreased following menthol treatment as compared to vehicle or baseline. These changes are indicative of allodynia following menthol treatment. At 24 and -4°C there were no significant effects following menthol treatment, and there were no significant differences between the sexes following menthol treatment.

Given the significant effect of menthol at 10°C for inducing cold sensitivity, we decided post-hoc to further investigate this difference by examining the change in success ratio over time at 10°C (37°C is also shown for comparison). We were interested in evaluating if there was a signature temporal pattern to this behavioral outcome. Recall that at 10°C there was a significant decrease in the success ratio relative to 37°C (corresponding to increased failure to lick, which is comparable to escape) when averaged for the entire trial (Fig. 3B). After almost 10 minutes of enhanced licking relative to the 10°C baseline, menthol substantially reduced the rate of licks per contact during the remainder of the 10°C trial period (Fig. 3D).

## Discussion

While many pain patients complain of heightened cold sensitivity, understanding the underlying mechanisms has proved to be difficult due to current limitations in translatable animal behavioral outcome measures. We recently developed an operant assay that can provide information regarding several dimensions of an animal's evaluation of thermal stimuli that are not addressed by traditional, reflex-based tests. Our goals in this study were two fold: we wanted to characterize behavioral responses to facial cold stimulation and evaluate the effects of menthol on cold sensitivity using an operant testing paradigm. We previously showed a significant decrease in operant behavioral outcome measures with increasingly hot temperatures in male rats under normal and inflammatory conditions [6]. We found in the present study that increasingly cold temperatures did not produce the dramatic reduction in behavioral outcome measures previously observed for heat. For facial stimulation we did not see a significant effect on outcome measures until the temperature was reduced to -4°C. Cold temperatures are not immediately damaging to tissue as is noxious heat and animals may be able to tolerate and overcome any initial discomfort from cold when motivated by food reinforcement.

Mauderli *et al*. [4] and Vierck *et al*. [5] have assessed escape latency and duration to a range of cold temperatures in an operant assay and compared them to typical reflex and innate measures. Escape latency for a range of cold temperatures (0.3–20°C) was much shorter than latencies to lick or guard, indicating that thresholds differed substantially for reflex and operant responses to cold. Also the duration of time spent off the stimulus platform (escape duration) increased with decreasing temperature. In contrast, there was little effect of temperature on time spent guarding or licking. Thus, reflex measures of cold nociception reveal different patterns of responsivity than do operant tests. Although we did not evaluate time away from the thermode, our findings are similar in that changes in escape responses to increasing cold and success ratios were modest for both operant tasks, especially when compared with the rate of response to increasing heat. While operant responding was not significantly hindered by moderate cold in the present study, we observed that the rats exhibited an increase in isolated facial grooming following contact with these temperatures, as previously described in models of orofacial hypersensitivity that measure unlearned behaviors [12].

Human psychophysical studies have revealed that responses to thermal stimulation are not merely reflective of the physical experience but also reflect the emotional state and views of the subject [13-15]. Cold stimulation in particular produces a more varied qualitative response in subjects than noxious heat, which is primarily described as a "burning" sensation [16]. These factors contribute to the experience of thermal pain in both normal and injured individuals. Traditional behavioral tests in animals are often unable to evaluate affective aspects of pain processing that are just as crucial as the physical transduction of thermal stimuli, and these procedures often introduce additional stress which can confound results [17]. This is likely the reason that some potential analgesics may seem promising in traditional animal studies, but fail during clinical trials.

In humans, sex differences with respect to cold pain tolerance and ratings have been noted [18,19]. It has been suggested that such difference may be due to psychosocial factors [20], although there is some evidence that circulating female hormones may mediate adaptation to cold pressor pain [21] and sex differences in cortisol may effect response to repeated cold stimulation [22]. It is known clinically that more women than men suffer from neuropathic pain [23,24] and that a common symptom of individuals with such pain is increased cold sensitivity [25]. Few studies have addressed behavioral responses to cold stimuli in animals and thus far none have looked at sex differences in sensory processing of cold stimuli for healthy animals in either a reflex based assay or an operant assay. In this study, we wanted to complete a general survey of the effects of sex and cold on operant measures. We observed significant differences between the sexes only at 10°C, with males exhibiting signs of increased sensitivity (greater facial contacts and decreased success ratio) as compared to females, which is in contrast to typical sex differences reported in human studies. However, the lack of significance at all other temperatures and outcomes indicates that the difference demonstrated here was not robust in healthy rats. Sex differences may be more pronounced with sustained stimulation [26]. Since animals self stimulate in this assay, the length of stimulation may not have been sufficient to reveal sex differences. This is certainly an area that merits further exploration.

In order to more directly compare cold and hot mediated behaviors, we used a thermal preference paradigm. Rats were given a choice between nociceptive cold (-4°C) and hot (48°C) stimulation, which produced nearly identical pain indices (reward licks/stimulus contact ratio and stimulus duration/contact ratio). This indicated that the magnitudes of pain sensations elicited by these stimuli were similar (Fig. 2D, E). However, when given the option to choose between these nociceptive hot or cold stimuli, rats spent more time on the hot side. Interestingly, this thermal preference test may reflect a greater influence of the affective component of cold as compared to heat pain of comparable sensory magnitude. This result is similar to human psychophysical ratings of hot and cold stimuli. Rainville *et al*. found that the relative unpleasantness, determined as a ratio of pleasantness ratings to perceived intensity, was greater for tonic (5°C) cold pressor stimulation than for phasic contact heat [27]. Greenspan *et al*. observed that for hot stimuli the initial temperature rated as unpleasant was within 1.4°C of the temperature rated as painful, but for cold stimuli unpleasantness ratings began at temperatures 5.6°C higher than the temperature rated as painful [28]. These findings indicate that in humans cool temperatures are perceived as more unpleasant than warm temperatures of a comparable magnitude, as we demonstrated in rats. This strong affective component to perception of cold stimuli may make it difficult to fully isolate the sensory component of cold perception.

Different exposure conditions lead to different sensory experiences, especially for cold stimuli, presumably due to activation of different populations of receptors and the influence of sympathetic activation on thermal sensations. In human subjects, repeated stimulation of glabrous skin with a cooled probe produces a deep radiating ache over time that increases in proportion to the decrease in superficial and deep skin temperature [29]. This effect is presumed to be due to activation of nociceptors near vessels. In addition, burning cold is evoked by temperatures near freezing and is attributed to activation of C-fibers that respond to both hot and cold stimuli [30]. It is likely that our operant paradigm involving numerous episodes of cold stimulation elicits both sensations.

Several populations of peripheral afferents have been identified that are responsive to cold and express different TRP family channels. TRPM8 is a receptor expressed on sensory neurons that has been identified as cold activated, although there is debate about its role in the processing of nociceptive cold stimulation [31-34]. In an attempt to address this issue, we examined the use of the mint derived substance, menthol, which acts as a specific agonist to the TRPM8 receptor. Following treatment with menthol, there was a significant increase in facial contacts and significant decreases in the ratio of licks to facial contacts and the duration of facial contacts at 10°C, indicating the induction of sensitization and development of allodynia.

The finding that successful completion of the operant task was hindered by menthol in the presence of 10 but not in the presence of 24°C provides evidence that activation of the TRPM8 receptor elicits sensations of cold pain. TRPM8 mRNA has been identified in both small and medium diameter DRG neurons *in vivo*, which are presumed to correspond to the cell bodies of C- and Aδ fibers, respectively. It is likely that both fiber types with TRPM8 receptors represent subpopulations of cold responsive nociceptors [35]. However, 24°C may not be of sufficient magnitude to activate TRPM8-containing C-fibers but could activate cold responsive Aδ fibers that block C-fiber activity and prevent the induction of menthol-induced allodynia [36].

Within the spinal dorsal horn, cool-sensitive lamina I spinothalamic (STT) cells have a specific sensitivity to temperatures between 34 and 15°C. The sensitivity of polymodal nociceptive HPC (for heat, pinch, cold) cells to noxious cold begins at about 24°C, and their response to cold accelerates at temperatures below 15°C. It has been suggested that an increase in HPC activity beyond that of cool-sensitive cells signals the sensation of burning pain [37]. Even in the presence of menthol it seems 24°C does not provide input of sufficient magnitude to increase HPC STT cell activity beyond cool-sensitive STT cells in lamina I and thus could explain why menthol did not enhance sensitivity to 24°C.

We had expected that menthol would cause cold hyperalgesia at -4°C, but we did not observe a significant difference when comparing the different treatments. Menthol is known to produce a burning sensation in humans at a concentration of 40% and also increases sensitivity to cold, presumably by its action on C-fibers [7]. It is possible that 10% menthol while sufficient to induce allodynia at 10°C, was not sufficiently concentrated to induce hyperalgesia at -4°C. It is important to note that it also did not induce an insensitivity to -4°C, ruling out the possibility that TRPM8 expressing cells were desensitized to further cold stimulation. It is also possible that sensations produced by -4°C stimulation are mediated more so by a population of cells expressing another putative cold transducing receptor, such as TRPA1 [31], or another as yet unidentified receptor [38,39].

## Conclusion

It seems clear that like heat, there are many molecular players involved in peripheral transduction of cold sensations. Additionally, higher order processing of cold sensations cannot be ignored. Thus, it is important to use behavioral tests that require engagement of higher order processing, and synthesize information regarding multiple dimensions of pain. Understanding how these varying aspects of the pain experience interact *in vivo *will help understand the regulation of molecular mechanisms of spontaneous and induced cold allodynia in humans. This understanding should ultimately lead to more effective treatments for patients suffering from pain.

## Methods

### General

Male and female hairless Sprague-Dawley rats (seven weeks old, Charles River, Raleigh, NC) were housed in groups of three to four in enriched housing, were maintained in a standard 12-hour light/dark cycle and were allowed access to food and water *ad libitum *when not being tested. We chose to use both males and females as there is evidence for sex related differences in thermal sensitivity for operant testing of the hindpaw (*Vierck personal communication*). Rats' weights were recorded every week to monitor general health. Females' estrus cycles were monitored by examination of vaginal lavage cytology as described in [40]. Animal testing procedures and general handling complied with the ethical guidelines and standards established by the Institutional Animal Care & Use Committee at the University of Florida, and all procedures complied with the Guide for Care and Use of Laboratory Animals (Council 1996).

### Behavioral Facial Testing

Facial testing was completed using a reward-conflict operant testing paradigm as described previously [6]. Briefly, the rats were trained to drink sweetened condensed milk while making facial contact with a thermode. During the training period (approximately 2 weeks) their baseline intake was recorded, and the rats were considered ready for experimental testing once their average baseline intake was 10 g or greater at 37°C. The facial testing region for each animal was depilitated under light isofluorane anesthesia (inhalation, 2.5 %) once a week to maximize thermal stimulus contact. The rats were fasted over night (13–15 hrs) prior to each session and were tested at 37, 24, 10, and -4°C on separate days.

Thermal preference of the animals was recorded using a modified testing box that had thermodes and sipper tubes situated side by side in a single compartment, with the rats free to access either reward tube equally (Fig. 2A). The rats (n = 7) were previously trained and tested in the single thermode boxes at 37, -4, and 48°C. Rats were initially placed in the thermal preference apparatus with both thermodes set at 37°C to allow them to become accustomed to this new task. A second such session was recorded to ensure rats did not demonstrate a side preference. In the third and fourth sessions one thermode was set cold (-4°C ± 1°C) and the other was set hot (48 ± 0.2°C). Rats were able to move freely from one side of the compartment to the other and explore both thermodes at will.

For the single thermode test, six outcome measures were evaluated as described previously [6]: reward intake (g), number of licking contacts, number of facial contacts with the thermode, total duration of facial contacts, ratio of licking contacts (reward) to facial contacts (stimuli), and the ratio of total duration of contacts/number of facial contacts were averaged over the 30 min trial periods. The ratio of licks to thermode contacts was also calculated as a function of time for each trial. For the thermal preference task, the first four outcome measures (intake, licking contacts, facial contacts, and duration of facial contacts) were calculated for each side of the box and totaled to determine the percentage of each outcome spent on each side and at each temperature. The ratio of licking contacts (reward) to facial contacts (stimuli) and the ratio of total duration of contacts/number of facial contacts were calculated for 37, -4, and 48°C. One and two-way ANOVAs were used to compare behavioral changes across different temperatures and treatments using SPSS (v. 14.0, SPSS, Inc.). When significant differences were found, post-hoc comparisons were made using Tukey's test. For the thermal preference task, a paired t-test was used to compare the percentage of time spent on the hot and the cold thermodes. A probability level of P < 0.05 was considered significant.

### Menthol and Vehicle Treatment

Menthol (10%) or vehicle (1.6% EtOH/0.01% Tween 80 in PBS) was delivered by subcutaneous injection (150 μl) into the cheeks of gently restrained rats. Rats were returned to their holding cages for 15 minutes prior to facial testing at -4°C, 10°C, or 24°C as described above. A cross-over design was used for drug administration whereby animals received both treatments at each temperature on separate testing days.

## Competing interests

The author(s) declare that they have no competing interests.

## Authors' contributions

HR was responsible for overseeing the behavioral testing, injecting the animals, analyzing and interpreting the data, and drafting the manuscript. CV and RC helped to draft and revise the manuscript. JN conceived the study, participated in its design, assisted with data analysis and interpretation, and helped to draft the manuscript. All authors read and approved the final manuscript.
